# Low Concentration of S100A8/9 Promotes Angiogenesis-Related Activity of Vascular Endothelial Cells: Bridges among Inflammation, Angiogenesis, and Tumorigenesis?

**DOI:** 10.1155/2012/248574

**Published:** 2012-05-17

**Authors:** Changyou Li, Siyuan Li, Changkai Jia, Lingling Yang, Zicheng Song, Yiqiang Wang

**Affiliations:** Shandong Provincial Key Laboratory of Ophthalmology, Shandong Eye Institute, Shandong Academy of Medical Sciences, Qingdao 266071, China

## Abstract

Previous studies showed that several members of the S100A family are involved in neovascularization and tumor development. This study checked whether low concentrations of S100A8 or S100A9 has any effect on the behaviour of vascular endothelial cells. A human umbilical vascular endothelial cell (HUVEC) line was used to measure vascular endothelial cell bioactivity related to angiogenesis, such as cell proliferation, migration, and vessel formation. In the low concentration range up to 10 **μ**g/mL, either each alone or in combination, S100A8 and S100A9 proteins promoted proliferation of HUVEC cells in a dose-dependent manner. The presence of both proteins in culture showed additive effects over each single protein. Both proteins enhanced HUVEC cells to migrate across the transwell membrane and to form tube-like structures on the Matrigel surface. When mixed in Matrigel and injected subcutaneously in Balb/c mice, both proteins increased vessel development in the gel plugs. Microarray assay of HUVEC cells treated with 10 **μ**g/mL S100A8 revealed that ribosome pathway, pathogenic *Escherichia coli* infection pathway, apoptosis, and stress response genes were modulated by S100A8 treatment. We propose that S100A8 and S100A9 proteins from either infiltrating inflammatory cells or tumor cells play an important role in the interplay among inflammation, angiogenesis, and tumorigenesis.

## 1. Introduction

While angiogenesis is fundamental to embryonic development and regeneration of injured tissues, unwanted angiogenesis, which is usually referred to as neovascularization, is a common pathological process of diseases such as cancer, autoimmune disorders, and transplant rejection. In recent years, the triangular relationship among inflammation, angiogenesis, and tumor development in the fields of cancer biology and immunology have been studied extensively, and the most encouraging progress is the gradual uncovering of the molecular mechanisms for inflammation-associated tumorigenesis [1–5]. In brief, many of the key molecules or pathways that are previously proven vital for inflammation or immunity, such as Nod1 [6], IKK*β* [7], SOCS3 [8], nitric oxide [9], TLR-MyD88 pathway [10], epigenetics [11, 12], or even T-cell activation [13] are now shown to be involved in inflammation-associated tumorigenesis, though the very first step at molecular level for inflammation-induced neoplastic transformation is yet to be determined. Once transformation succeeds and neoplatic cells appear, the interplay between inflammation and tumor development becomes more complex and dynamic in determining the fate of the transformed cells [14, 15], and sooner or later, another player, namely, angiogenesis, will join. On one side, a large number of cytokines, chemokines, or enzymes produced by inflammatory cells modulate tumor cells growth or the formation of blood vessels in tumor mass. On the other side, tumor cells may secrete some molecules that attract and modulate inflammatory cells. Thus it is feasible to control tumors by targeting neovascularization [16, 17], or by interfering the inflammation-tumor process [18] or the inflammation-neovascularization crosstalk [19].

 In a preliminary research project using chemical burn- or suture-induced inflammatory corneal neovascularization models, we identified two well-documented inflammation mediators mainly produced by infiltrating neutrophils in such models, namely, S100A8 and S100A9, as potential promoters of neovascularization [20]. When looking into the potential mechanisms for such activity of S100A8/A9, we found that low concentrations of S100A8/A9 promoted proliferation, migration, and tube formation of vascular endothelial cells. Taking into account the fact that many tumors produce S100A8/A9 to a certain extent [21–23], we proposed that S100A8/A9, from either tumor cells or infiltrating leukocytes, promote the transformed cells to create a blood vessel supply for themselves.

## 2. Materials and Methods

### 2.1. Cell and Proliferation Assay

Human umbilical vascular endothelial cells (HUVECs) were maintained in Dulbecco's modified Eagle's medium (DMEM) supplemented with 10% heat-inactivated fetal bovine serum. Cell proliferation was measured by using 3-(4,5-dimethyl-2-thiazolyl)-2,5-diphenyl-2H-tetrazolium bromide (MTT) assay. In brief, HUVEC were seeded in 96-well microplates at 5 × 10^4^ cells/100 *μ*L/well, cultured overnight in complete culture medium, washed with and starved in serum-free DMEM for 6 hours, and then treated with recombinant low-endotoxin grade human S100A8 or S100A9 (Cyclex Co. Ltd., Nagano, Japan) at 0, 1, 5, and 10 *μ*g/mL in serum-free DMEM for 24 hours. Twenty microliters of 5 mg/mL MTT reagent (Sigma, St. Louis, MO) was added to each well, and the incubation continued for another 4 hours. After removal of the medium containing the MTT reagent, 150 *μ*L dimethyl sulfoxide were added to each well. The plates were shaken for 10 min before reading by a MD2 microplate reader (Molecular Devices, Sunnydale, CA) at a wavelength of 490 nm.

### 2.2. Cell Migration Assay

Cell migration was assessed by using transwell membrane inserts with 8.0 *μ*m pores (Corning, Acton, MA). Five hundred microliters of culture medium with or without S100A8 (10 *μ*g/mL), S100A9 (10 *μ*g/mL), or S100A8 + S100A9 protein (10 *μ*g/mL both) were placed in the lower chambers, and 4 × 10^5^ HUVEC in 200 *μ*L complete medium were added to the upper chamber. The plate was incubated at 37°C for 4 hours and the filters were then removed for fixation in methanol. Cells that migrated to the lower surface of the filter were stained with 0.1% crystal violet and counted using a light microscope in four random fields per well. The results are expressed as the number of migrated cells per field. Three inserts were set for each group in this experiment.

### 2.3. *In Vitro* Cell Vascular Tube Formation Assay

*In vitro* tube formation assays was carried out according to previously reported methods, with modifications. The wells of a 96-well tissue culture plate were coated with 40 *μ*L of ice-cold Growth Factor Reduced Matrigel (BD Bioscience, San Jose, CA) at 37°C for 1 hour. Twenty thousand HUVEC in 200 *μ*L medium with or without S100A8, S100A9, or S100A8 + S100A9 proteins were added to each well. After incubation for 6 hours at 37°C in 5% CO_2_, the cultures were photographed and the tube-like structures were evaluated.

### 2.4. *In Vivo* Matrigel Plug Angiogenesis Assay

The Matrigel implantation assay was performed as outlined by McMahon et al. [24]. In brief, 300 *μ*L of Matrigel supplemented with S100A8, S100A9, or both to 10 *μ*g/mL were injected subcutaneously into the ventral groin region of Balb/c mice (Beijing Institute of Pharmacology, Beijing, China). The same volume of Matrigel alone, or supplemented with 200 ng/mL fibroblast growth factor (FGF) (R&D, Minneapolis, MN) plus heparin (10 U/mL), was used as negative or positive controls, respectively. After 10 days, Matrigel implants were isolated from the surrounding tissues, washed with PBS, and photographed. Each specimen was divided evenly into two pieces, one being fixed for routine histopathology, the other frozen and lyophilized overnight. The dried Matrigel was weighed, rehydrated in 0.4 mL of 0.1% Triton X-100 for 1 hour, disrupted by vigorous pipetting, and centrifuged at 14,000 ×g for 15 min to remove particulates. The concentration of hemoglobin in the solution was determined by comparing the absorbance at 405 nm to a standard curve made with purified hemoglobin (Sigma). Use of animals was approved by the Shandong Eye Institute Review Board with permit number SEIRB-2009-2009CB526506 in accordance with the Guidelines on the Humane Treatment of Laboratory Animals (Chinese Ministry of Science and Technology, 2006) and the Association for Research in Vision and Ophthalmology (ARVO) Statement for the Use of Animals in Ophthalmic and Vision Research.

### 2.5. Gene Profiling Using Microarray Assay

HUVECs were seeded in six-well microplates at 6 × 10^4^ cells/1.5 mL/well. S100A8 was added to 10 *μ*g/mL, and untreated wells were included as control for S100A8 treatment, both in triplicates. After 4 or 24 hours of culture, the cells were harvested and total RNA was prepared by using QIAGEN RNeasy Mini Kit (Qiagen Inc., Valencia, CA) as recommended by the manufacturer. The integrity and high quality of RNA samples were confirmed by using agarose electrophoresis. Two micrograms of RNA were reverse-transcribed to cDNA, during which process a T7 sequence was introduced into the cDNA. T7 RNA polymerase-driven RNA synthesis was used for the preparation and labeling of cRNA with Cy3 (the S100A8-treated samples) and Cy5 (the control samples), respectively. Equal amounts (875 ng) of purified Cy3- and Cy5-labelled probes were mixed and used for hybridisation on one Agilent Human Oligo 4x44K Microarray (Agilent Technologies Inc., Santa Clara, CA) following the protocol provided by the manufacturer. This type of microarray covers 41,000+ unique genes and transcripts. Three independent pairs of samples for each time point were utilized on three microarrays, respectively. The hybridization signals were acquired by using an Agilent G2565BA Microarray Scanner System and analyzed using Agilent G2567AA Feature Extraction Software 10.0 (Agilent Technologies, Santa Clara, CA) with default settings. Specifically, the Linear Lowes dye method was used for normalization of the features. Based on the global normalization, each feature was marked by the analysis software as absent (A), marginal (M), or present (P). To be counted as P, a feature has to pass four criteria, namely: (1) it is positive and significant versus the background, (2) the signals are uniform in the spot, (3) the signals are not saturated, and (4) there are no population outliers in either channel. To minimise the risk of false positives when evaluating the expression change, only those probes that had one or less “A” and gave average signal densities above 200 for the three replicate arrays were subjected to further analysis. The differentially expressed genes at the two time points were annotated and compared using the Database for Annotation, Visualization and Integrated Discovery (DAVID, v6.7) with the whole human genome as the background [25]. The complete sets of raw and normalized data of this microarray assay are deposited in the NCBI Gene Expression Omnibus (GEO) with the GEO accession number GSE33768.

## 3. Results

### 3.1. S100A8 and S100A9 Promote HUVEC Proliferation

**Figure 1:**
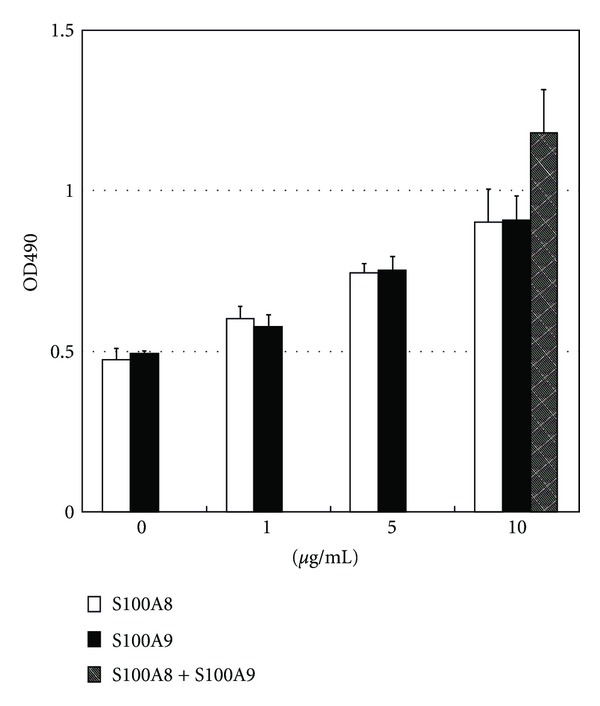
Stimulation of HUVEC proliferation following S100A8 or S100A9 protein treatment at different concentrations. Shown are representatives of three experiments with similar results.

When added separately to culture medium at 1, 5, and 10 *μ*g/mL, both S100A8 and S100A9 showed dose-dependent stimulatory effects on HUVEC proliferation. When both S100A8 and S100A9 were present at 10 *μ*g/mL, a moderate additive effect was noticed (Figure 1). This indicates that, though S100A8 and S100A9 were proposed to form heterodimers (S100A8/A9) under physiological conditions, each of them alone also manifested biological activity on HUVEC. In the following studies, we utilized 10 *μ*g/mL as a representative concentration of both proteins.

### 3.2. S100A8 and S100A9 Stimulate Migration and Tube Formation of HUVEC

**Figure 2:**
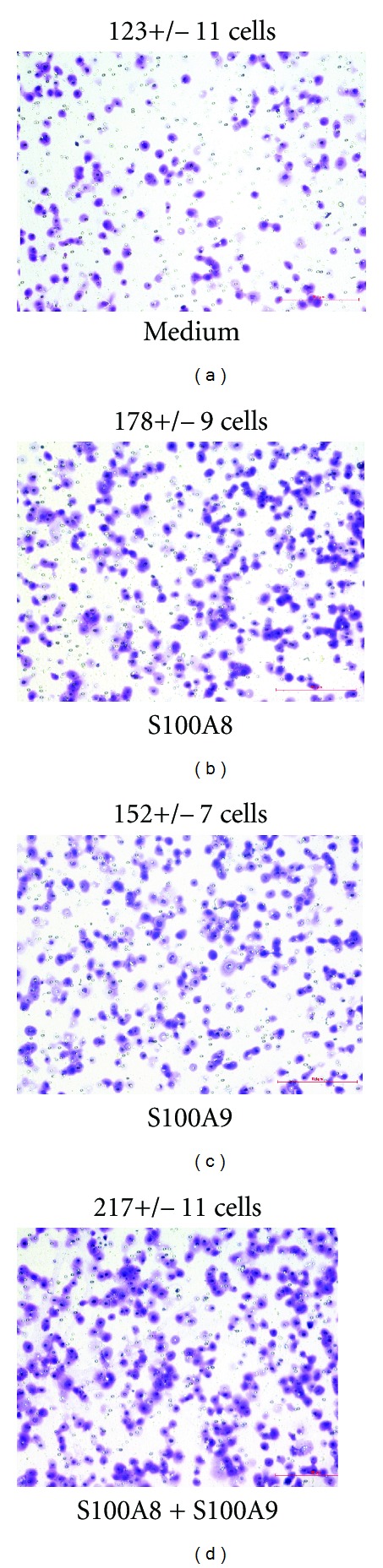
Photographs of HUVEC cells migrated through transwell chambers. The numbers above each panel are the cell counts per microscope field (mean ± standard deviation, *n* = 6). *P < 0.05 versus medium control by the two-tailed, paired Student's *t*-test.

**Figure 3:**
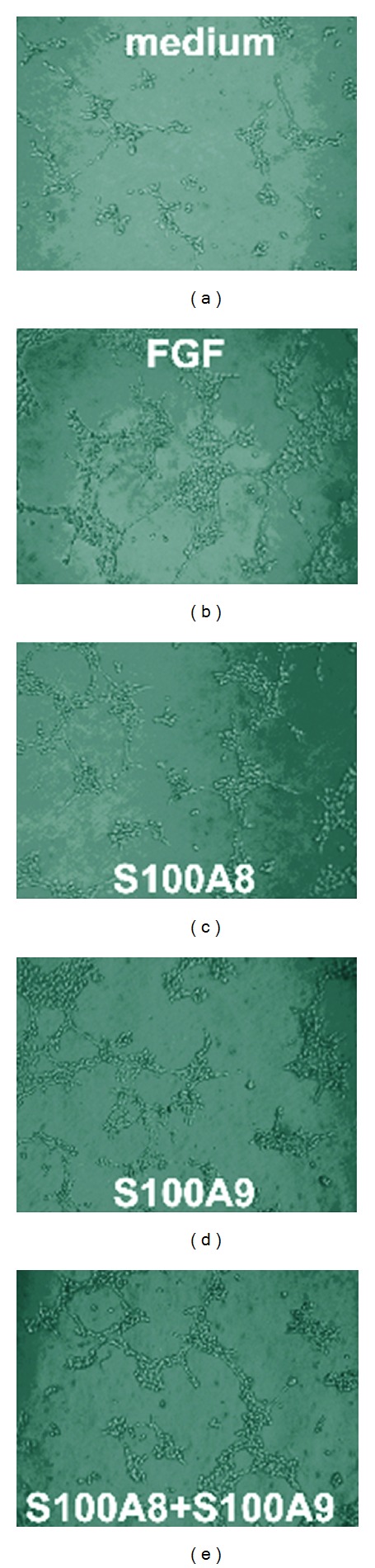
Tube-like structure formation of HUVEC seeded on Matrigel with or without S100A8 or S100A9 protein *in vitro* (both at 10 *μ*g/mL). FGF plus heparin was used as a positive control.

A transwell chamber system was employed to measure the effect of S100A8 and S100A9 proteins on endothelial cell migration or invasion. As shown in Figure 2, S100A8, S100A9, or S100A8 + S100A9 increased HUVEC migration across the transwell membrane. Similarly, S100A8 and S100A9 proteins also promoted tube-like structure formation of cultured HUVEC on Matrigel (Figure 3). While S100A9 was less effective than S100A8 in both readouts, an additive effect for S100A8 and S100A9 was also observed.

### 3.3. S100A8 and S100A9 Proteins Promote Vascularization *In Vivo*

**Figure 4:**
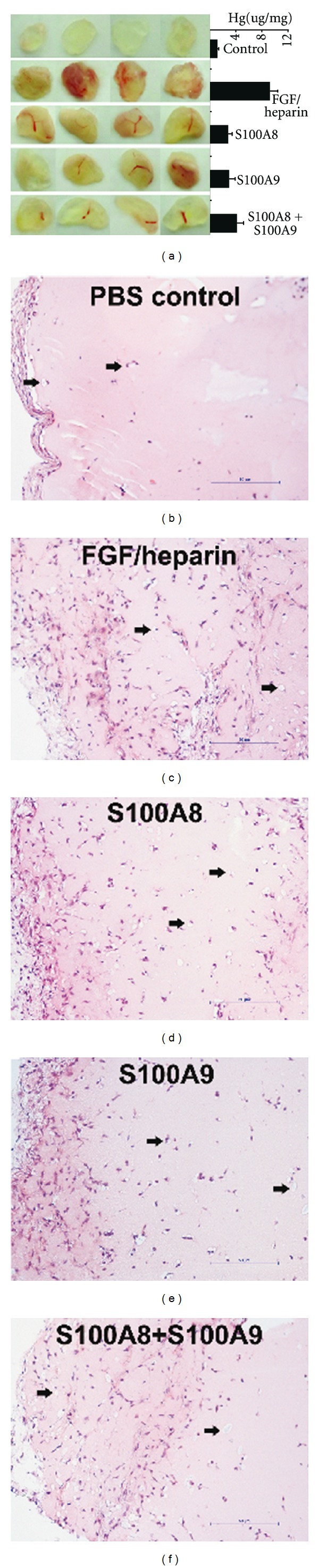
Photographs (pictures, upper left panel), haemoglobin contents (solid columns, upper left panel), and histology of Matrigel plugs indicating increased neovascularization following S100A8 or S100A9 protein treatment. Matrigel mixed with PBS was the negative control group, and FGF plus heparin was the positive control group. Haemoglobin content of Matrigel plugs (mean ± standard deviation, *n* = 4) was expressed as the amount of haemoglobin per gram of Matrigel plug. Microscopic examination of the same Matrigel plugs showed inflammatory cells and vascular structures (arrowheads). Shown are representations of two experiments with similar results.

Matrigel plug assay *in vivo* was used to assess the proangiogenic activity of S100A8, S100A9, or S100A8 plus S100A9. On the tenth day after injection of Matrigel premixed with the tested proteins, plugs were removed for analysis. A representative implant for each treatment group is shown in Figure 4. Gross examination of the plugs, hemoglobin measurement, and histological study indicated that S100A8, S100A9, or their combination significantly enhanced blood vessel formation in the plugs, but their effects at 10 *μ*g/mL were significantly lower than that of 200 ng/mL FGF plus 10 U/mL heparin.

### 3.4. Ribosome Pathway and Pathogenic *Escherichia coli* Infection Pathway Were Modulated by S100A8 Treatment

Lastly, we profiled the whole-genome gene expression patterns in HUVEC to screen for the genes or pathways responsible for the effect of low concentrations of S100A8. After culture in 10 *μ*g/mL S100A8 for 4 or 24 hours, 189 probes in total were regulated by over 1.5-fold. Among these probes, 34 did not relate to any defined genes, while the other 155 probes corresponded to 143 defined genes in total (Table S1 of the supplementary material available online at doi:10.1155/2012/248574). While many genes showed a concerted change (either upregulation or downregulation) at both time points, no gene showed an opposite change at these two time points (Tables S1 and S2). For those genes having two or three probes in this array, the changes of the probes were consistent with each other, such as with metallothionein 2A (Table 1). This also reflects the reliability and accuracy of the microarray results. DAVID analysis of the changed genes revealed that ribosome pathway-related genes were enriched over 30-fold in the downregulated genes in both conditions (Table S3, Figure S1). Another main pathway negatively modulated by S100A8 treatment was pathogenic *Escherichia coli* infection (PECI) (Table S3). In more detail, the four downregulated genes involved in the PECI pathways at 4 hours were beta actin (ACTB, NM_001101), gamma 1 actin (ACTG1, NM_001614), keratin 18 (KRT18P19, NM_199187), and alpha 1c tubulin (TUBA1C, NM_032704). The four genes downregulated at 24 hours were ACTB, ACTG1, KRT18P19, and beta 2C tubulin (TUBB2C, NM_006088). Due to the limitation of the available numbers of the upregulated genes, no pathways were shown to be significantly upregulated.

### 3.5. Apoptosis and Stress Response Genes Were Modulated by S100A8 Treatment

Continuing along the ontology of the altered genes, it was found that most of the enriched GO terms are closely related with cellular metabolism, for example, transcription, translation, apoptosis, ribosome biogenesis, and so on (Table S4). In terms of apoptosis, nine genes were downregulated at 4 hours after S100A8 treatment. They were KRT18P19; lectin, galactoside-binding, soluble, 1 (LGALS1, NM_002305); nonmetastatic cells 1, protein (NM23A) expressed in (NME1-NME2, and NM_198175); nucleophosmin 1 (nucleolar phosphoprotein B23, numatrin) pseudogene 21 (Npm1, NM_006993); protein phosphatase 3 (formerly 2B), regulatory subunit B, alpha isoform (Ppp3r1, NM_000945); ribosomal protein S3A pseudogene 5 (RPS3AP5, XM_001719310); transmembrane protein 102 (Tmem102, NM_178518); ubiquitin B (UBB, NM_018955); ubiquitin C (UBC, NM_021009). At 24 hours, four apoptosis-related genes were downregulated, including RPS3AP5, TUBB2C, UBB, and UBC. Several genes that were of special interest are listed in Table 1. For example, S100A6 was downregulated by S100A8 treatment, while none of the other members of the S100A family showed any change (Table S5). On the contrary, none of the genes that were reported to respond to high concentrations (200 *μ*g/mL) of S100A8 in vascular endothelial cells [26, 27] showed any significant changes in this study (Table S6).

## 4. Discussions

We previously reported that neutralization of S100A8 using specific monoclonal antibody inhibited vessel development in experimental inflammatory corneal neovascularization [20]. Now, by measuring the direct effect of S100A8, and S100A9 proteins on HUVEC, we showed that these two proteins, when present at low concentrations, promote angiogenesis. This is contrary to observations that high concentrations of S100A are pro-apoptotic to vascular endothelial cells [26, 27]. Thus, the study described here extends our knowledge about the interplay among inflammation, angiogenesis, and tumorigenesis. While other members of this family, such as S100A4 [28, 29], S100A7 [30] and S100A13 [31], have been shown to participate under similar conditions, this discussion focuses on S100A8/A9's role in tumorigenesis by reviewing the effects of S100A8/A9 on tumor cells or vascular endothelial cells. Table S7 summarizes the main reports concerning expression changes of S100A8, S100A9, or S100A8/A9 in tumors versus normal corresponding tissues. While most studies showed that S100A8/A9 is overexpressed in various types of cancers [23, 32, 33], these proteins might also be underexpressed in some other cancers [34]. As with the effect of S100A8/A9 on cell growth, apparently contradictory observations exist. Some *in vitro* studies have demonstrated the apoptosis-inducing effects of S100A8/A9 in tumor cells [35, 36]. A recent study demonstrated that low concentrations of S100A8/A9, namely, 5 or 10 *μ*g/mL, significantly promoted tumor cell proliferation of human breast cancers and human neuroblastoma cell lines; when the concentration of S100A8/A9 was increased to 25 *μ*g/mL, however, the promoting effect disappeared [37]. Interestingly enough, those authors showed that the promotion effect of low concentrations of S100A8/A9 on tumor cells proliferation was mediated by the RAGE-NF*κ*B pathway, a pathway that was proposed in other studies to mediate the pro-apoptotic effect of S100A8/A9 [38, 39].

On the other hand, S100A8/A9 has long been known to regulate vascular inflammation. To the best of our knowledge, all existing reports proposed that S100A8/A9 serves as an injury signal for endothelial cells of the vascular endothelium, mainly via promoting leukocyte recruitment [40] and inducing proinflammatory responses in endothelial cells [26, 27]. In a recent study using *in vitro* endothelial cells culture in combination with oligonucleotide microarray profiling, it was shown that treatment of human microvascular endothelial cells with 200 *μ*g/mL heterodimeric S100A8/A9 resulted in an upregulation of several genes that are known to promote platelet aggregation, inflammation, and endothelial permeability [27]. Interestingly, none of these genes showed significant change in our current study (Table S6). On the contrary, our array assay suggested the metabolism-related genes or pathways to be the main responders to 10 *μ*g/mL S100A8 treatment. Correspondingly, our study is also the first one to show that low concentrations of S100A8 and S100A9, either alone or together, stimulate proliferation, migration, and vascular formation of endothelial cells.

**Figure 5:**
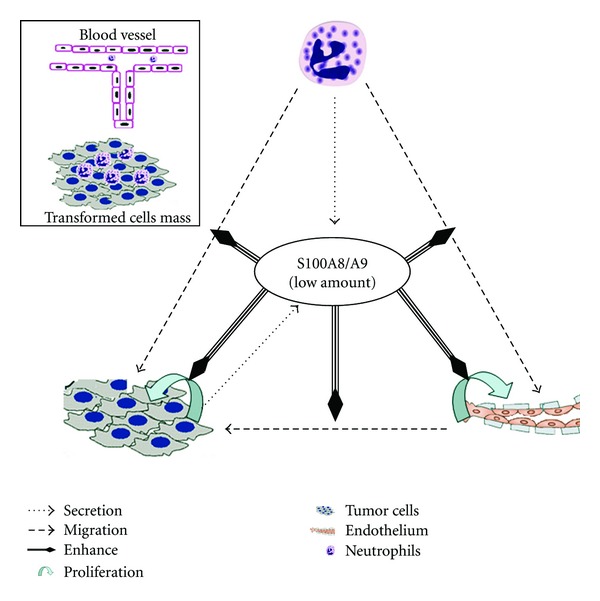
Roles of S100A8/A9 in tumorigenesis. In this hypothesis, S100A8/A9 are produced by newly formed tumor cells or infiltrating leukocytes. When they reach adequately low concentrations, they can stimulate (1) tumor cells proliferation, (2) vascular endothelial cell proliferation, migration, and formation of vascular structures, and (3) neutrophils to migrate into tumor mass or adhere to endothelium. By producing S100A8/A9, the newly entering neutrophils enhance local inflammation and neovascularization in turn. Other S100A family members like S100A4, S100A7, and S100A13 may join S100A8 and/or S100A9 in this episode as well.

This study is of significance, since it proposes a new explanation for why inflammation could be tumorigenic or why small vascular-free tumor cell clusters develop blood vessels and then keep on growing. We propose that S100A8/A9, and perhaps also other S100A proteins, either produced by tumor cells or by infiltrating inflammatory cells [41], promote neovascularization in tumor mass by promoting endothelial cells behavior (Figure 5). In this hypothesis, especially at the early stage of cell transformation or tumor formation, the S100A8/A9 produced from whatever cells, start to accumulate locally and reach a range that allow them to stimulate tumor cells or endothelial cells proliferation, migration, and so forth, thus favor growth of mass tumor. Our hypothesis partially explains why certain chronic inflammation is tumorigenic, and why expression of S100A8/A9 in tumors could be regarded as a prognostic marker of several types of tumors [22, 42]. So far no data are available with the levels of S100A8/A9 in tumor mass, but we predict that their levels might never be high enough to cause pro-apoptotic effects on either tumor cells or endothelial cells. This prediction was supported by several studies. For example, in a study that proposed that serum S100A9 would serve as a useful marker to discriminate between prostate cancer and benign prostatic hyperplasia, the serum S100A9 concentration measured by ELISA was about 2–14 ng/mL in cancer patients [22]. The predicted enhancer effect of S100A8/A9 on tumor development was also confirmed by a couple of experimental studies. Németh et al. reported that increasing S100A8/A9 expression in mouse hepatocellular carcinoma cells via transfection protected cells from death and resulted in malignant progression [43]. Similarly, over-expression of S100A8 in keratinocytes via adenoviral transduction protected cells from irradiation-induced apoptosis [44]. Considering the fact that other S100A family members like S100A4, S100A7, and S100A13 are also involved in angiogenesis as well as inflammation, this hypothesis are potentially applicable with the whole S100A family.

In conclusion, our results suggest that S100A8 and S100A9 proteins at relatively low concentrations have the potential to promote angiogenesis through directly enhancing proliferation, migration, and tube formation of vascular endothelial cells. This finding not only provides a new explanation for neovascularization development in situations of inflammation or tumor, but also suggests novel targets during the management of related diseases such as tumors. Future studies would investigate into the details of production and action of these inflammation mediators in the context of the interplays between tumor cells and vascular endothelial cells.

## Supplementary Material

The supplemental materials mainly contains the analysis results of our own microarray data (Fig S1, Table S1-S5). In brief, Table S1 lists all genes that were up- or down-regulated by 1.5 folds or over in at least one time point after treatment with 10ug/mL S100A8. Table S2 is the category of such gene according to their expression change. Table S3 lists the pathways of the genes that changed upon S100A8 treatment, while Figure S1 depicts the down-regulated genes that belong to the Ribosome pathways. Table S4 lists the representatives of altered GOs upon S100A8 treatment. Table S5 details the expression levels of S100A family members in S100A8-treated HUVEC compared with control cells. Besides, Table S6 compared the cell cycle-related gene expression in current study (with 10*μ*g/mL S100A8 only) to that of Viemann studies (with 200*μ*g/mL S100A8/A9. Blood 2005, 105:2955-62. Blood 2007, 109:2453-60). Finally, Table 7 summarizes the main findings about expression changes of S100A8, S100A9 or S100A8/A9 in tumors versus normal corresponding tissues.

## Figures and Tables

**Table 1:** Representative of changed genes upon S100A8 treatment.

Genbank accession	Gene name	4 hr	24 hr
NM_005497	gap junction protein, gamma 1, 45 kDa	1.724 ± 0.307	0.971 ± 0.089
NM_003714	stanniocalcin 2	1.453 ± 0.014	1.507 ± 0.168
NM_000584	interleukin 8	1.404 ± 0.246	1.738 ± 0.189
NM_002133	heme oxygenase (decycling) 1	1.686 ± 0.425	1.881 ± 0.266
NM_014624	S100 calcium binding protein A6	0.418 ± 0.043	0.552 ± 0.036
NM_002422	matrix metallopeptidase 3	0.952 ± 0.094	1.557 ± 0.104
NM_005950	metallothionein 1G	0.589 ± 0.021	0.654 ± 0.039
NM_005952	metallothionein 1X	0.690 ± 0.033	0.789 ± 0.044
NM_005953	metallothionein 2A	0.530 ± 0.057	0.624 ± 0.100
NM_005953	metallothionein 2A	0.421 ± 0.054	0.492 ± 0.058
NM_005953	metallothionein 2A	0.417 ± 0.045	0.470 ± 0.039
