# Supplementary material for: Low Concentration of S100A8/9 Promotes Angiogenesis-Related Activity of Vascular Endothelial Cells: Bridges among Inflammation, Angiogenesis, and Tumorigenesis?
Source: Mediators Inflamm. 2012 May 17;2012:248574. doi: 10.1155/2012/248574 (PMC3363068; doi:10.1155/2012/248574)
Supplement: Supplementary file 1 — The supplemental materials mainly contains the analysis results of our own microarray data (Fig S1, Table S1-S5). In brief, Table S1 lists all genes that were up- or down-regulated by 1.5 folds or over in at least one time point after treatment with 10ug/mL S100A8. Table S2 is the category of such gene according to their expression change. Table S3 lists the pathways of the genes that changed upon S100A8 treatment, while Figure S1 depicts the down-regulated genes that belong to the Ribosome pathways. Table S4 lists the representatives of altered GOs upon S100A8 treatment. Table S5 details the expression levels of S100A family members in S100A8-treated HUVEC compared with control cells. Besides, Table S6 compared the cell cycle-related gene expression in current study (with 10μg/mL S100A8 only) to that of Viemann studies (with 200μg/mL S100A8/A9. Blood 2005, 105:2955-62. Blood 2007, 109:2453-60). Finally, Table 7 summarizes the main findings about expression changes of S100A8, S100A9 or S100A8/A9 in tumors versus normal corresponding tissues. [file 248574.f1.pdf]

Table S1. Folds of genes expression change in HUVEC after treatment with 10ug/mL S100A8

Probe [a,b]	RefSeq	Gene description	fold-4hr	fold-24hr
A_23_P120883	NM_002133	heme oxygenase (decycling) 1	1.686	1.881
A_24_P418517	AL353580	nucleophosmin pseudogene	1.746	1.746
A_24_P59239			1.751	1.723
A_24_P170186			1.724	1.744
A_24_P178154			1.716	1.678
A_24_P58759	AC008628	similar to chaperonin containing Tcp1, subunit 5 (epsilon)	1.651	1.670
A_24_P33429	AADB02001452	similar to nucleophosmin 1	1.635	1.680
A_24_P621434			1.605	1.709
A_24_P358337			1.687	1.595
A_24_P814872			1.635	1.598
A_24_P350160	NM_198262	arginine/serine-rich coiled-coil 2	1.690	1.543
A_24_P306921	AADB02017686	similar to ATP synthase, H+ transporting, mitochondrial F0 complex, subunit B1	1.629	1.591
A_24_P332862	NM_175910	zinc finger protein 493	1.642	1.561
A_24_P202497	NM_020648	twisted gastrulation homolog 1 (Drosophila)	1.565	1.548
A_24_P209378	NM_016220	zinc finger protein 107	1.553	1.519
A_24_P281801			1.533	1.526
A_24_P32849	NM_001011725	heterogeneous nuclear ribonucleoprotein A1-like 2	1.764	
A_32_P165477	NM_014331	solute carrier family 7, (cationic amino acid transporter, y+ system) member 11		1.755
A_32_P24376	NM_033184	keratin associated protein 2-1; keratin associated protein 2-4; keratin associated protein 2-3; similar to keratin associated protein 2-4; keratin associated protein 2-2		1.742
A_32_P87013	NM_000584	interleukin 8		1.738
A_23_P253622	XM_001719592	similar to KIAA1641; similar to ankyrin repeat domain 26; ankyrin repeat domain 36B	1.725	
A_23_P428287	NM_001080383	gap junction protein, gamma 1, 45kDa	1.724	
A_24_P187094				1.723
A_24_P307075	AC097709	similar to heterogeneous nuclear ribonucleoprotein A1	1.708	
A_23_P142272	NM_019088	Paf1, RNA polymerase II associated factor, homolog (S. cerevisiae)	1.706	
A_24_P717305			1.699	
A_32_P24382	NM_033184	keratin associated protein 2-1; keratin associated protein 2-4; keratin associated protein 2-3; similar to keratin associated protein 2-4; keratin associated protein 2-2		1.683

A_24_P273284			1.660	
A_23_P317620	NM_005737	ADP-ribosylation factor-like 4C	1.649	
A_32_P28365	NM_152298	nuclear autoantigenic sperm protein (histone-binding)	1.644	
A_24_P16892	NM_003184	TAF2 RNA polymerase II, TATA box binding protein (TBP)-associated factor, 150kDa	1.625	
A_32_P126467	BX537549	hypothetical LOC645784	1.612	
A_23_P53557	NM_002342	lymphotoxin beta receptor (TNFR superfamily, member 3)	1.611	
A_24_P58187	XM_062025	similar to heterogeneous nuclear ribonucleoprotein A1-like	1.608	
A_24_P521662				1.604
A_24_P229807			1.604	
A_24_P256764	NM_005964	myosin, heavy chain 10, non-muscle	1.587	
A_23_P501634	NM_078476	butyrophilin, subfamily 2, member A1		1.585
A_24_P42316	NM_018429	B double prime 1, subunit of RNA polymerase III transcription initiation factor IIIB	1.585	
A_23_P4014	NM_017575	Smg-6 homolog, nonsense mediated mRNA decay factor (C. elegans)	1.583	
A_32_P56525	NM_014719	family with sequence similarity 115, member A; family with sequence similarity 115, member B (pseudogene)	1.580	
A_24_P541482				1.579
A_32_P83326	AC139495	hypothetical LOC100272216	1.578	
A_24_P150486	NM_004863	serine palmitoyltransferase, long chain base subunit 2	1.577	
A_32_P207054	NM_013986	similar to Ewing sarcoma breakpoint region 1; Ewing sarcoma breakpoint region 1	1.576	
A_24_P247536	NM_152789	family with sequence similarity 133, member B pseudogene; similar to FAM133B protein; family with sequence similarity 133, member B		1.569
A_24_P409904	AK130705	hypothetical LOC100272216	1.568	
A_32_P181513	XM_001719792	zinc finger protein 730	1.565	
A_23_P103996	NM_002061	glutamate-cysteine ligase, modifier subunit		1.561
A_23_P161698	NM_002422	matrix metallopeptidase 3 (stromelysin 1, progelatinase)		1.557
A_23_P50834	NM_182515	zinc finger protein 714	1.553	
A_24_P41662				1.552
A_32_P221958	NM_001077685	ArfGAP with GTPase domain, ankyrin repeat and PH domain 7		1.552
A_24_P324405	XM_001720760	ankyrin repeat domain 11; hypothetical	1.545	

		protein LOC100128265		
A_24_P288993	NM_001168331	hypothetical gene supported by AF044957; NM_004547; NADH dehydrogenase (ubiquinone) 1 beta subcomplex, 4, 15kDa		1.541
A_32_P82409				1.537
A_24_P131785	NM_003704	chromosome 4 open reading frame 8	1.535	
A_24_P41530			1.531	
A_32_P216426			1.528	
A_23_P44643	NM_001137664	anaphase promoting complex subunit 7	1.526	
A_32_P148345	NM_004039	annexin A2 pseudogene 3; annexin A2; annexin A2 pseudogene 1	1.524	
A_24_P891276	CH236948	hypothetical LOC442292	1.518	
A_24_P384059	AL450226	heterogeneous nuclear ribonucleoprotein A1pseudogene	1.517	
A_32_P116556	NM_001127464	zinc finger protein 469		1.515
A_24_P101601			1.512	
A_24_P418536	AL359915	ribosomal protein L6 pseudogene 2	1.511	
A_24_P187355			1.510	
A_24_P33525			1.510	
A_32_P209735	BI907421	glucuronidase, beta-like 2; glucuronidase, beta pseudogene	1.510	
A_24_P290188				1.509
A_23_P206822	NM_015171	exportin 6		1.509
A_23_P416395	NM_003714	stanniocalcin 2		1.507
A_24_P204238	XR_078725	similar to Huntingtin interacting protein K		1.507
A_24_P312189	NM_019606	methylphosphate capping enzyme	1.501	
A_24_P889720	NM_021009	ubiquitin C	0.636	0.657
A_24_P681218			0.642	0.610
A_23_P60933	NM_005950	metallothionein 1G	0.589	0.654
A_23_P90143	NM_012423	ribosomal protein L13a pseudogene 7; ribosomal protein L13a pseudogene 5; ribosomal protein L13a pseudogene 16; ribosomal protein L13a; ribosomal protein L13a pseudogene 18	0.610	0.624
A_32_P151544	NM_199187	keratin 18; keratin 18 pseudogene 26; keratin 18 pseudogene 19	0.589	0.620
A_24_P142223	NM_033251	ribosomal protein L13 pseudogene 12; ribosomal protein L13	0.585	0.624
A_23_P217609	NM_001032393	ribosomal protein L36a pseudogene 51; ribosomal protein L36a pseudogene 37; ribosomal protein L36a pseudogene 49; heterogeneous nuclear ribonucleoprotein H2	0.549	0.653

		(H'); ribosomal protein L36a		
A_24_P315986			0.551	0.613
A_23_P166459	NM_002305	lectin, galactoside-binding, soluble, 1	0.526	0.637
A_23_P46182	NM_001012	ribosomal protein S8; ribosomal protein S8 pseudogene 8; ribosomal protein S8 pseudogene 10	0.515	0.646
A_32_P208178	XM_001719310	ribosomal protein S3A pseudogene 5; ribosomal protein S3a pseudogene 47; ribosomal protein S3a pseudogene 49; ribosomal protein S3A; hypothetical LOC100131699; hypothetical LOC100130107	0.509	0.650
A_23_P252413	NM_005953	metallothionein 2A	0.530	0.624
A_32_P11931	NM_000987	ribosomal protein L26 pseudogene 33; ribosomal protein L26; ribosomal protein L26 pseudogene 16; ribosomal protein L26 pseudogene 19; ribosomal protein L26 pseudogene 6	0.524	0.627
A_24_P681301	NM_021009	ubiquitin C	0.553	0.581
A_32_P857658	NM_001003	ribosomal protein, large, P1	0.515	0.616
A_24_P754803	NM_006013	ribosomal protein L10; ribosomal protein L10 pseudogene 15; ribosomal protein L10 pseudogene 6; ribosomal protein L10 pseudogene 16; ribosomal protein L10 pseudogene 9	0.565	0.558
A_23_P120660	NM_001024	ribosomal protein S21	0.495	0.626
A_23_P423419	NR_022014	chromosome 15 open reading frame 21	0.528	0.552
A_32_P137939	NM_001101	actin, beta	0.508	0.565
A_23_P147888	NM_001004	ribosomal protein, large, P2 pseudogene 3; ribosomal protein, large, P2	0.478	0.589
A_32_P190488	AC078817	ribosomal protein L26 pseudogene 32	0.461	0.605
A_24_P142228	NM_033251	ribosomal protein L13 pseudogene 12; ribosomal protein L13	0.521	0.524
A_32_P175198	NM_001614	actin, gamma 1	0.484	0.545
A_24_P135902	NM_002952	ribosomal protein S2 pseudogene 8; ribosomal protein S2 pseudogene 11; ribosomal protein S2 pseudogene 5; ribosomal protein S2 pseudogene 12; ribosomal protein S2 pseudogene 51; ribosomal protein S2 pseudogene 17; ribosomal protein S2 pseudogene 55; ribosomal protein S2 pseudogene 20; ribosomal protein S2	0.437	0.571

A_32_P184796	NM_053275	ribosomal protein, large, P0 pseudogene 2; ribosomal protein, large, P0 pseudogene 3; ribosomal protein, large, P0 pseudogene 6; ribosomal protein, large, P0	0.507	0.499
A_24_P922631	NM_001102609	hypothetical protein LOC133874	0.461	0.515
A_23_P201711	NM_014624	S100 calcium binding protein A6	0.418	0.552
A_24_P148235	NM_001030	ribosomal protein S27 pseudogene 29; ribosomal protein S27 pseudogene 9; ribosomal protein S27 pseudogene 23; ribosomal protein S27 pseudogene 13; ribosomal protein S27; ribosomal protein S27 pseudogene 21; ribosomal protein S27 pseudogene 7; ribosomal protein S27 pseudogene 6; ribosomal protein S27 pseudogene 19	0.438	0.524
A_24_P179339			0.499	0.450
A_24_P113109	NM_018955	ubiquitin B	0.484	0.460
A_23_P331028	NM_015335	mediator complex subunit 13-like	0.446	0.490
A_23_P106708	NM_002952	ribosomal protein S2 pseudogene 8; ribosomal protein S2 pseudogene 11; ribosomal protein S2 pseudogene 5; ribosomal protein S2 pseudogene 12; ribosomal protein S2 pseudogene 51; ribosomal protein S2 pseudogene 17; ribosomal protein S2 pseudogene 55; ribosomal protein S2 pseudogene 20; ribosomal protein S2	0.418	0.513
A_23_P106844	NM_005953	metallothionein 2A	0.421	0.492
A_24_P361896	NM_005953	metallothionein 2A	0.417	0.470
A_24_P179336			0.454	0.426
A_23_P37445	NM_213725	ribosomal protein, large, P1	0.401	0.470
A_23_P135084	NM_000972	ribosomal protein L7a pseudogene 70; ribosomal protein L7a; ribosomal protein L7a pseudogene 30; ribosomal protein L7a pseudogene 66; ribosomal protein L7a pseudogene 27; ribosomal protein L7a pseudogene 11; ribosomal protein L7a pseudogene 62	0.405	0.421
A_24_P323805			0.353	0.451
A_23_P214046	NM_033644	F-box and WD repeat domain containing 11	0.665	
A_32_P49728	XM_002342218	hypothetical protein LOC339803	0.664	
A_24_P188071	NM_032704	tubulin, alpha 1c	0.662	

A_32_P342064	NM_002032	ferritin, heavy polypeptide 1; ferritin, heavy polypeptide-like 16; similar to ferritin, heavy polypeptide 1; ferritin, heavy polypeptide-like 3 pseudogene	0.659	
A_23_P368205	NM_001135637	phosphatidylinositol-4-phosphate 5-kinase, type I, alpha		0.658
A_24_P366989			0.658	
A_24_P582241			0.657	
A_32_P94798	NM_004039	annexin A2 pseudogene 3; annexin A2; annexin A2 pseudogene 1		0.657
A_23_P97283	NM_198406	progestin and adipoQ receptor family member VI		0.655
A_32_P112380	NM_178518	transmembrane protein 102	0.655	
A_23_P38167	NM_022036	G protein-coupled receptor, family C, group 5, member C		0.654
A_32_P53670			0.653	
A_23_P59179	NM_021976	retinoid X receptor, beta		0.653
A_23_P141389	NM_000988	ribosomal protein L27	0.652	
A_23_P77779	NM_000981	ribosomal protein L19; ribosomal protein L19 pseudogene 12	0.652	
A_32_P231391				0.650
A_32_P41487	XM_001725257	hypothetical LOC729505; similar to hCG2040565; high-mobility group nucleosomal binding domain 2; similar to high-mobility group nucleosomal binding domain 2		0.649
A_24_P144025	NM_001024	ribosomal protein S21	0.648	
A_23_P208991	NM_002579	paralemmin		0.648
A_23_P65307	NM_032229	SLIT and NTRK-like family, member 6		0.648
A_23_P400181	NR_026052	hypothetical LOC65996		0.647
A_24_P134074	NM_001022	ribosomal protein S19 pseudogene 3; ribosomal protein S19	0.647	
A_24_P14485	NM_017495	RNA binding motif protein 38	0.646	
A_23_P208358	NM_001136134	ribosomal protein L28	0.645	
A_23_P68240	XM_002348280	hypothetical protein LOC150763; similar to hCG1732629; similar to glycerol-3-phosphate acyltransferase, mitochondrial	0.645	
A_23_P79323	NM_003936	cyclin-dependent kinase 5, regulatory subunit 2 (p39)	0.644	
A_24_P258235	NM_001004739	olfactory receptor, family 5, subfamily L, member 2	0.643	
A_32_P24794			0.642	
A_23_P218675	NM_006103	WAP four-disulfide core domain 2		0.641

A_24_P272590	AC008446	ribosomal protein S15 pseudogene 6	0.641	
A_23_P4679	NM_006494	Ets2 repressor factor	0.640	
A_23_P38085	NM_001042476	calcium regulated heat stable protein 1, 24kDa		0.637
A_24_P763243	NM_001402	eukaryotic translation elongation factor 1 alpha-like 7; eukaryotic translation elongation factor 1 alpha-like 3; similar to eukaryotic translation elongation factor 1 alpha 1; eukaryotic translation elongation factor 1 alpha 1		0.636
A_23_P101532	NM_001015	ribosomal protein S11 pseudogene 5; ribosomal protein S11	0.634	
A_24_P58881			0.633	
A_23_P18292	NM_003973	ribosomal protein L14	0.632	
A_32_P93782	NM_000987	ribosomal protein L26 pseudogene 33; ribosomal protein L26; ribosomal protein L26 pseudogene 16; ribosomal protein L26 pseudogene 19; ribosomal protein L26 pseudogene 6	0.632	
A_23_P141549	NM_001011	ribosomal protein S7; ribosomal protein S7 pseudogene 11; ribosomal protein S7 pseudogene 4; ribosomal protein S7 pseudogene 10	0.629	
A_23_P106204	NM_145870	glutathione transferase zeta 1	0.626	
A_24_P418418	NM_001021	ribosomal protein S17	0.620	
A_23_P70127	NM_017510	transmembrane emp24 protein transport domain containing 9		0.616
A_24_P273666	NM_016592	GNAS complex locus		0.614
A_23_P315286	NM_138774	chromosome 19 open reading frame 22	0.613	
A_23_P374149	NM_015050	FtsJ methyltransferase domain containing 2		0.612
A_24_P204144			0.611	
A_23_P85171	NM_000117	emerin	0.611	
A_24_P188941	XM_496355	nucleophosmin 1 (nucleolar phosphoprotein B23, numatrin) pseudogene 21; hypothetical LOC100131044; similar to nucleophosmin 1; nucleophosmin (nucleolar phosphoprotein B23, numatrin)	0.609	
A_23_P64090	NM_030981	RAB1B, member RAS oncogene family	0.606	
A_32_P36884	CH471143	FK506 binding protein 1C	0.605	
A_23_P117721	NM_001021	ribosomal protein S17	0.605	
A_23_P104188	NM_004433	E74-like factor 3 (ets domain transcription factor, epithelial-specific)		0.603

A_24_P213783	NM_001099693	ribosomal protein L31 pseudogene 49; ribosomal protein L31 pseudogene 17; ribosomal protein L31	0.599	
A_24_P160001	NM_000801	FK506 binding protein 1A, 12kDa	0.599	
A_23_P325093	NM_178026	gamma-glutamyltransferase 7		0.597
A_23_P157405	XM_946272	coiled-coil-helix-coiled-coil-helix domain containing 2; similar to coiled-coil-helix-coiled-coil-helix domain containing 2	0.595	
A_24_P418239	NM_001025071	ribosomal protein S14	0.595	
A_23_P33045	NM_000987	ribosomal protein L26 pseudogene 33; ribosomal protein L26; ribosomal protein L26 pseudogene 16; ribosomal protein L26 pseudogene 19; ribosomal protein L26 pseudogene 6	0.591	
A_23_P38167			0.591	
A_23_P141405	NM_198175	non-metastatic cells 1, protein (NM23A) expressed in; NME1-NME2 readthrough transcript; non-metastatic cells 2, protein (NM23B) expressed in	0.588	
A_23_P30464	NM_030567	proline rich 7 (synaptic)	0.583	
A_23_P24763	NM_001017	ribosomal protein S13 pseudogene 8; ribosomal protein S13; ribosomal protein S13 pseudogene 2	0.580	
A_23_P104318	NM_019058	DNA-damage-inducible transcript 4		0.579
A_23_P128067	NM_021104	ribosomal protein L41	0.575	
A_24_P388252	NM_000945	protein phosphatase 3 (formerly 2B), regulatory subunit B, alpha isoform	0.567	
A_23_P6433	NM_005368	myoglobin		0.567
A_23_P251593	NM_007104	ribosomal protein L10a pseudogene 6; ribosomal protein L10a; ribosomal protein L10a pseudogene 9	0.564	
A_23_P29747	NM_000984	ribosomal protein L23a pseudogene 63; ribosomal protein L23a pseudogene 75; ribosomal protein L23a pseudogene 37; ribosomal protein L23a pseudogene 65; ribosomal protein L23a pseudogene 43; ribosomal protein L23a pseudogene 44; ribosomal protein L23a	0.559	
A_23_P502274	NM_002751	mitogen-activated protein kinase 11	0.557	
A_23_P208925	NM_003025	SH3-domain GRB2-like 1	0.556	
A_23_P142475	NM_001031	ribosomal protein S28 pseudogene 6; ribosomal protein S28 pseudogene 9;	0.546	

		ribosomal protein S28		
A_23_P81492	NM_001025071	ribosomal protein S14	0.536	
A_32_P220307	NM_001000	ribosomal protein L39 pseudogene 10; ribosomal protein L39 pseudogene 20; ribosomal protein L39 pseudogene 27; ribosomal protein L39; ribosomal protein L39 pseudogene 13; ribosomal protein L39 pseudogene 32	0.536	
A_32_P187327	NM_006088	tubulin, beta 2C		0.530
A_23_P252322	NM_001001977	ATP synthase, H+ transporting, mitochondrial F1 complex, epsilon subunit pseudogene 2; ATP synthase, H+ transporting, mitochondrial F1 complex, epsilon subunit	0.512	
A_32_P75141				0.498
A_23_P33759	NM_004753	dehydrogenase/reductase (SDR family) member 3		0.496
A_23_P52298	NM_006993	nucleophosmin/nucleoplasmin, 3	0.477	

Note: [a] Agillent probe ID in 4X44 microarray.

[b] The probes/genes are basically listed in the order of change folds in either group.

Table S2. Summary of the altered probes or their corresponding genes

	Up-regulated genes			down-regulated genes		
	**only at 4hr**	**At both 4hr and 24hr**	**Only at 24hr**	**only at 4hr**	**At both 4hr and 24hr**	**Only at 24hr**
probes	37	16	20	54	38	22
Defined genes	29	10	13	44	27	20

Table S3. Altered pathways upon S100A8 treatment*

	Count	(%)	P value	Fold enrichment
**Up-regulated at 4hr or 24hr**				
none				
**Downregulated at 4hr (total 2)**				
Ribosome	29	42.0	9.47E-41	37.7
Pathogenic Escherichia coli infection	4	5.8	0.01	7.93
**Downregulated at 24hr (total 4)**				
Ribosome	14	28.6	3.66E-17	30.31
Pathogenic Escherichia coli infection	4	8.2	0.003	13.22
Vibrio cholerae infection	3	6.1	0.033	10.09
Dilated cardiomyopathy	3	6.1	0.080	6.14

Table S4. Representatives of altered GOs upon S100A8 treatment [a]

	Count	(%)	P value	Fold enrichment
**Up-regulation at 4hr (total 13)**				
regulation of transcription	9	22.5	0.026	2.23
in utero embryonic development	3	7.5	0.027	10.98
blood vessel morphogenesis	3	7.5	0.038	9.16
positive regulation of signal transduction	3	7.5	0.070	6.55
secretion	3	7.5	0.072	6.44
skeletal system development	3	7.5	0.080	6.06
cardiac muscle tissue development	2	5.0	0.081	22.60
positive regulation of cell communication	3	7.5	0.084	5.87
**Up-regulation at 24hr (total 1)**				
response to oxidative stress	3	12.5	0.009	19.04
**Downregulation at 4hr (total 55)**				
translational elongation	32	46.4	3.33E-52	71.43
ribosome biogenesis	11	15.9	1.06E-10	20.33
rRNA processing	8	11.6	1.37E-07	19.61
ncRNA processing	8	11.6	1.60E-05	9.65
negative regulation of protein modification process	4	5.8	0.015	7.58
erythrocyte homeostasis	3	4.3	0.019	13.80
negative regulation of RNA splicing	2	2.9	0.022	90.19
long-term strengthening of neuromuscular junction	2	2.9	0.022	90.19
regulation of apoptosis	9	13.0	0.023	2.52
response to caffeine	2	2.9	0.034	56.37
cytokine-mediated signaling pathway	3	4.3	0.037	9.66
response to purine	2	2.9	0.043	45.09
negative regulation of cellular protein metabolic process	4	5.8	0.044	5.01
homeostatic process	8	11.6	0.044	2.40
positive regulation of binding	3	4.3	0.045	8.67
axonogenesis	4	5.8	0.052	4.67
positive regulation of protein ubiquitination	3	4.3	0.052	8.05
release of sequestered calcium ion into cytosol	2	2.9	0.059	32.21

homeostasis of number of cells	3	4.3	0.071	6.76
nucleoside diphosphate metabolic process	2	2.9	0.072	26.53
cellular di-, tri-valent inorganic cation homeostasis	4	5.8	0.077	3.97
cell morphogenesis involved in differentiation	4	5.8	0.090	3.70
**Downregulation at 24hr (total 32)**				
translational elongation	17	34.7	2.36E-24	54.21
axonogenesis	5	10.2	0.003	8.34
cell morphogenesis involved in neuron differentiation	5	10.2	0.004	7.71
cell morphogenesis involved in differentiation	5	10.2	0.006	6.60
cellular component morphogenesis	6	12.2	0.007	4.87
cell motion	6	12.2	0.014	4.07
long-term strengthening of neuromuscular junction	2	4.1	0.015	128.84
cell morphogenesis	5	10.2	0.022	4.52
muscle cell differentiation	3	6.1	0.052	7.99
ribosome biogenesis	3	6.1	0.053	7.92
ER-associated protein catabolic process	2	4.1	0.070	26.84
induction of apoptosis	4	8.2	0.072	4.03

[a]. For clarity and simplicity, only representative (i.e. the one with least P value) in each group of similar GO terms were given. For example, “blood vessel development” and “vasculature development” are similar to “blood vessel morphogenesis” and thus omitted from this list. Similarly, “regulation of apoptosis”, “regulation of programmed cell death”, “induction of programmed cell death” and “regulation of cell death” overlaps “induction of apoptosis” thus omitted in this table. For the whole lists of GO, please refer to supplementary Table x.

Table S5. Expression levels of S100A family members in S100A8-treated HUVEC compared with control cells

Probe	Gene	4hr Signal (mean)[a]		4hr Ratio[b]		24hr signal (mean)[a]		24hr Ratio[b]	
		Control	S100A8	Mean	Stdev	Control	S100A8	Mean	Stdev
A_23_P137984	S100A10	62774.1	64817.9	1.035	0.046	61219.6	69547.2	1.139	0.063
A_23_P126593	S100A11	20579.4	24018.0	1.168	0.083	21724.7	24256.9	1.117	0.112
A_23_P145863	S100A11	22441.6	23169.6	1.031	0.086	23787.3	23388.7	0.983	0.072
A_23_P372874	S100A13	44426.6	45256.3	1.021	0.080	42314.6	47599.2	1.126	0.050
A_23_P147918	S100A16	30449.6	30527.1	1.002	0.022	28849.5	28965.2	1.004	0.021
A_23_P201706	S100A2	12544.5	10876.3	0.868	0.052	7201.4	5968.9	0.832	0.054
A_23_P104073	S100A3	4291.4	3752.1	0.875	0.020	3466.9	3153.7	0.909	0.033
A_23_P94800	S100A4	347.1	295.2	0.852	0.067	291.6	257.2	0.883	0.065
A_23_P201711	S100A6	92629.2	38507.6	0.418	0.043	106115.7	58853.7	0.552	0.036
A_24_P222835	S100PBP	973.3	1028.6	1.058	0.048	1008.1	1070.9	1.065	0.115
A_23_P338952	S100PBP	1783.4	1719.3	0.966	0.076	1754.5	1780.0	1.014	0.082
A_23_P200425	S100PBP	2362.1	2174.5	0.920	0.030	2374.2	2248.8	0.946	0.92

a. Shown were mean of three arrays in each group. b.Mean and standard deviation of ratios in three arrays were give.

Table S6. Comparison of cell cycle-related gene expression in Viemann studies (with 200µg/mL S100A8/A9) (1, 2) to that of current study (with 10µg/mL S100A8 only)

**Genes up-regulated over 1.7 folds at 6 hr by S100A8/A9**	**Our data at 4 hr**	**Genes down-regulated over 1.7 folds at 6 hr by S100A8/A9**	**Our data at 4 hr**
Bak (Bcl-2—antagonist/killer 1) BAK1	1.320	ERCC5 (excision repair protein complementation group 5)	0.994
Bax (Bcl-2—associated X protein)	0.886, 0.790, 0.737	NEK3 (never in mitosis gene A–related kinase 3)	1.129, 1.036, 1.027
TP53 (Transcription factor p53)	0.943	MAD2L1 (MAD2 (mitotic arrest deficient 2)–like 1)	0.975
		CDC2 (Cell division cycle 2 (G1 to S and G2 to M))	1.018
		API5 (Apoptosis inhibitor 5)	1.033, 0.903
		NEK4 (NIMA-related kinase 4)	
		STAG2 (Stromal antigen 2)	1.028, 1.105
		CFLAR (c-FLIP (CASP8 and FADD-like apoptosis regulator)	0.968, 1.104
		CGRRF1 (Cell growth regulator with ring finger domain 1)	1.135, 1.019
		BBP (Beta-amyloid binding protein)	
		PMS1 (Postmeiotic segregation increased 1)	1.050
		CAST (Calpastatin)	1.143, 0.987
		RB1 (Retinoblastoma 1)	1.060, 1.032

Table S7. Summary of references concerning S100A8/A9 in tumors form patients or tumor cells lines

Tumor	Methods[a]	Main findings[b]	Ref.
**Positive relationship of S100A8/A9 expression with tumor**			
squamous cervical cancer (SCC)	2DE-MS, WB, IHC	S100A9 protein was highly up-regulated in SCC samples (by 2D-MS), found in all of 20 SCC tissues and 4 of 20 matched normal cervical tissues (by WB). Positive staining in all SSC samples but focal and weak staining in part of normal cervical tissues.	(3)
gastric cancer	MS	S100A8, A9 and α defensin 1, 2 were over-expressed in tumor biopsies compared with normal tissues.	(4)
invasive ductal carcinoma of the breast	IHC	S100A8 is S100A9-dependently expressed. Co-expression of both proteins was associated with poor tumor differentiation, vessel invasion, node metastasis, and advanced stage. Co-expression of the proteins was also observed in MCF-7 cells.	(5)
advanced stage ovarian cancer	2DE-MS, IHC	S100A8 was significantly up-regulated in pathological tissues and interstitial fluid. Weak S100A8 expression in epithelium but not stroma of normal ovarian tissues.	(6)
colorectal cancer (CRC) samples	2DE-MS, IHC, RT-PCR	S100A8 and S100A9 were over-expressed in both CRC tumor samples and serum. IHC showed that S100A8/A9 were mainly in tumor infiltrating immune cells rather than in tumor cells.	(7)
human breast tumor cell line and patients samples	MS, MA, IHC (tissue array)	S100A9 was over expressed in basal-like compared to luminal-like cell lines, and much more frequently expressed in basal-like tumors versus luminal-like tumors. Higher expression of S100A9 was associated with lower metastasis-free and overall survival rates.	(8)
non-muscle-invasive bladder cancer (NMIBC)	RT-qPCR	S100A8 may contribute to the generation of certain aspects of the aggressive phenotype rather than simply promoting cell proliferation in NMIBC.	(9)
muscle invasive bladder cancer (MIBC),	MA, RT-qPCR	Together with *IL1B* and *EGFR*, *S100A8 and S100A9 form a four-gene* indicator of tumor progression.	(10)
human prostate cancers	IHC, ISH, ELISA	S100A8, S100A9, and their potential receptor RAGE were up-regulated in prostatic intraepithelial neoplasia and preferentially in high-grade adenocarcinomas, whereas benign tissue was negative or showed weak expression of the proteins. There was a high degree of overlap of S100A8 and S100A9 expression patterns and of S100A8 or S100A9 and RAGE, respectively.	(11)
bladder cancer	HPLC,	S100A8 was over-expression more often in tumor with bladder	(12)

	2DE-MS, IHC	wall muscle invasion than in those without invasion. Abnormal expression of S100A8 and A9 are correlated with poor prognosis	
pancreatic adenocarcino ma	2DE-MS	Enhanced expression of S100A8, S100A9, and RAGE is an early event in prostate tumorigenesis and may contribute to development and progression or extension of prostate carcinomas. Furthermore, S100A9 in serum may serve as useful marker to discriminate between prostate cancer and BPH	(13)
chemical-indu ced mouse and rat bladder cancers, human bladder cancers	Array, RT-qPCR	Human cancers over-express S100A2, A3, A5, A7, A8, A9, A14, A15, A16 and S100P, and underexpress S100A1, A4 and S100B. S100A1, A3, A8, A9, A14, A15 and A16 showed similar change patterns in bladder cancers from mouse, rat and human.	(14)
21 common tumor types	Tissue array	A8 and A9 were expressed in 12% and 28% of breast cancers, respectively. S100A11 exclusively expressed in nuclear in normal tissues but translocated to cytoplasmic and nuclear in all common cancers.	(15)
**Negative relationship of S100A8/A9 expression with tumor**			
untransformed and isogenic human breast cancer cell lines including a normal, benign, noninvasive and invasive carcinoma.	MA, RT-PCR, WB	Down-regulation of TNFSF7, S100A4, S100A7, S100A8, and S100A9 (calcium binding protein family) were associated with transformation and progression of breast cancer cells. Exogenous expressions of S100A8 and S100A9 inhibit growth in benign and noninvasive carcinoma cells, suggesting their negative role in cell proliferation.	(16)
oral brush of normal, premalignant, and tumor cells	ProteinChi p Arrays, IHC	S100A8 and S100A9 distinguish between normal cells (high expression) and tumor cells (expression undetectable) with a sensitivity of 100% and specificity of 91% and can distinguish inflammatory/hyperproliferative lesions (intermediate expression) from tumor cells with a sensitivity of up to 91% and specificity of up to 90%.	(17)
human esophageal squamous cell carcinoma (ESCC) versus normal esophageal	RT-PCR	S100A1, A2, A4, A8, A9, A10, A11, A12, A14, S100B, and S100P genes were significantly down-regulated in ESCC while S100A7 markedly up-regulated	(18)

mucosa.			

a.2DE-MS, two dimension gel electrophoresis followed by mass spectrum of interested protein spors. WB, western blotting. IHC, immunohisotchemistry. RT-PCR, reverse transcription-PCR. RT-qPCR, RT-quantitative PCR. MA, microarray. ISH, in situ hybridization. HPLC, high performance liquid chromatography. b.some of the statements were directly copied from original references while others edited from references.

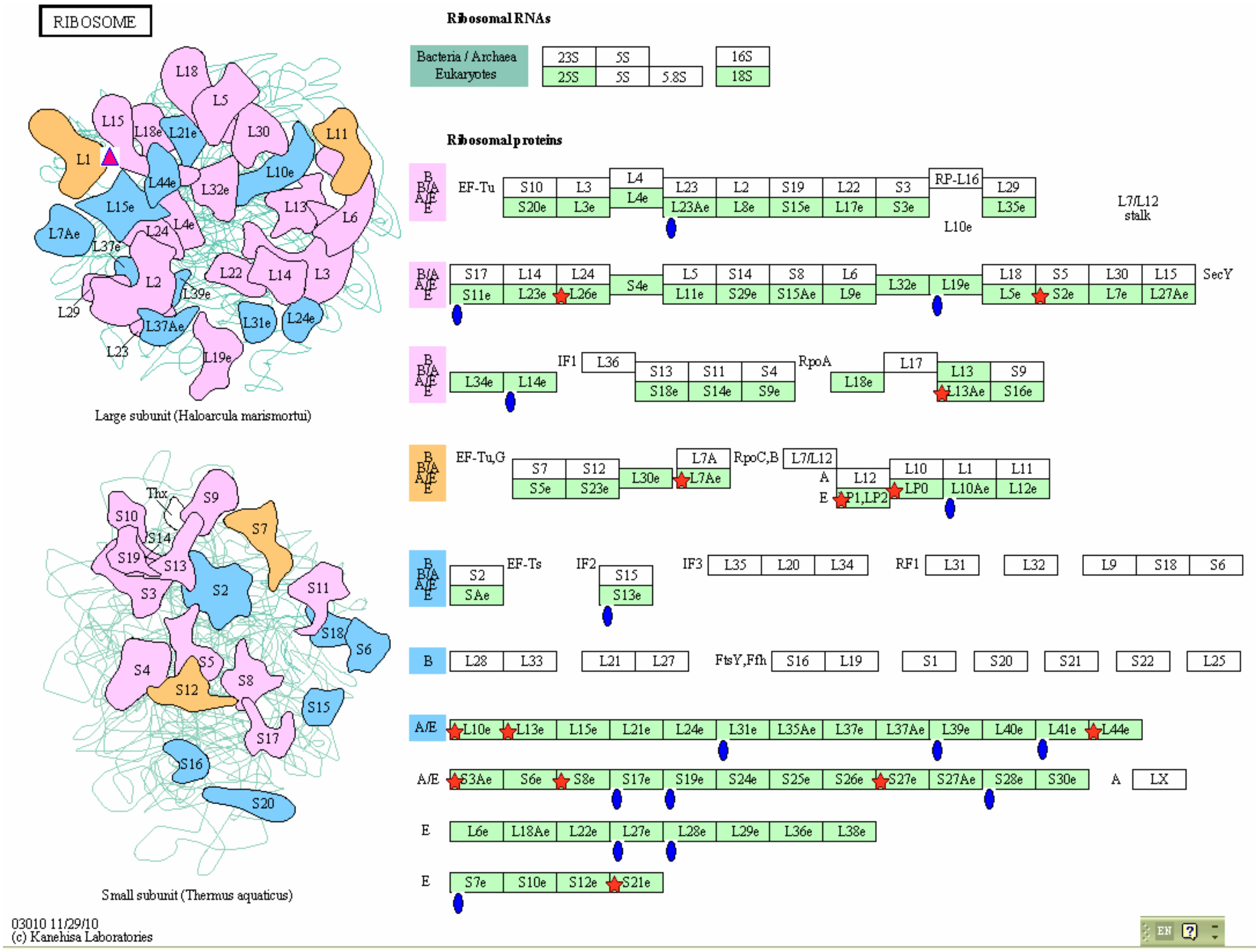

Figure S1. Downregulated genes that belong to the Ribosome pathways. Red stars indicate the genes that were downregulated at both 4 hr and 24 hr, blue ovals indicate the genes that were downregulated only at 4 hr. No genes were downregulated at 24 hr only without change at 4 hr.

Reference:

1. Viemann D, Strey A, Janning A, et al. Myeloid-related proteins 8 and 14 induce a specific inflammatory response in human microvascular endothelial cells. *Blood* 2005;**105**:2955-62.
2. Viemann D, Barczyk K, Vogl T, et al. MRP8/MRP14 impairs endothelial integrity and induces a caspase-dependent and -independent cell death program. *Blood* 2007;**109**:2453-60.
3. Zhu X, Lv J, Yu L, Wu J, Zou S, Jiang S. Proteomic identification of differentially-expressed proteins in squamous cervical cancer. *Gynecol Oncol* 2009;**112**:248-56.
4. Kim HK, Reyzer ML, Choi IJ, et al. Gastric cancer-specific protein profile identified using endoscopic biopsy samples via MALDI mass spectrometry. *J Proteome Res* 2010;**9**:4123-30.
5. Arai K, Takano S, Teratani T, Ito Y, Yamada T, Nozawa R. S100A8 and S100A9 overexpression is associated with poor pathological parameters in invasive ductal carcinoma of the breast. *Curr Cancer Drug Targets* 2008;**8**:243-52.
6. Cortesi L, Rossi E, Della Casa L, et al. Protein expression patterns associated with advanced stage ovarian cancer. *Electrophoresis* 2011;**32**:1992-2003.
7. Kim HJ, Kang HJ, Lee H, et al. Identification of S100A8 and S100A9 as serological markers for colorectal cancer. *J Proteome Res* 2009;**8**:1368-79.
8. Goncalves A, Charafe-Jauffret E, Bertucci F, et al. Protein profiling of human breast tumor cells identifies novel biomarkers associated with molecular subtypes. *Mol Cell Proteomics* 2008;**7**:1420-33.
9. Ha YS, Kim MJ, Yoon HY, et al. mRNA Expression of S100A8 as a Prognostic Marker for Progression of Non-Muscle-Invasive Bladder Cancer. *Korean J Urol* 2010;**51**:15-20.
10. Kim WJ, Kim SK, Jeong P, et al. A four-gene signature predicts disease progression in muscle invasive bladder cancer. *Mol Med* 2011;**17**:478-85.
11. Hermani A, Hess J, De Servi B, et al. Calcium-binding proteins S100A8 and S100A9 as novel diagnostic markers in human prostate cancer. *Clin Cancer Res* 2005;**11**:5146-52.
12. Minami S, Sato Y, Matsumoto T, et al. Proteomic study of sera from patients with bladder cancer: usefulness of S100A8 and S100A9 proteins. *Cancer Genomics Proteomics* 2010;**7**:181-9.
13. Shen J, Person MD, Zhu J, Abbruzzese JL, Li D. Protein expression profiles in pancreatic adenocarcinoma compared with normal pancreatic tissue and tissue affected by pancreatitis as detected by two-dimensional gel electrophoresis and mass spectrometry. *Cancer Res* 2004;**64**:9018-26.
14. Yao R, Lopez-Beltran A, Maclennan GT, Montironi R, Eble JN, Cheng L. Expression of S100 protein family members in the pathogenesis of bladder tumors. *Anticancer Res* 2007;**27**:3051-8.
15. Cross SS, Hamdy FC, Deloulme JC, Rehman I. Expression of S100 proteins in normal human tissues and common cancers using tissue microarrays: S100A6, S100A8, S100A9 and S100A11 are all overexpressed in common cancers. *Histopathology* 2005;**46**:256-69.
16. Rhee DK, Park SH, Jang YK. Molecular signatures associated with transformation and progression to breast cancer in the isogenic MCF10 model. *Genomics* 2008;**92**:419-28.
17. Driemel O, Murzik U, Escher N, et al. Protein profiling of oral brush biopsies: S100A8 and S100A9 can differentiate between normal, premalignant, and tumor cells. *Proteomics Clin Appl* 2007;**1**:486-93.
18. Ji J, Zhao L, Wang X, et al. Differential expression of S100 gene family in human esophageal squamous cell carcinoma. *J Cancer Res Clin Oncol* 2004;**130**:480-6.
